# Supplementary material for: HIVprotI: an integrated web based platform for prediction and design of HIV proteins inhibitors
Source: J Cheminform. 2018 Mar 9;10:12. doi: 10.1186/s13321-018-0266-y (PMC5845081; doi:10.1186/s13321-018-0266-y)
Supplement: Supplementary file 2 — Additional file 2. Source code of HIVProtI web server. [file 13321_2018_266_MOESM2_ESM.zip › HIVprotI_Source-code/hivprotI/weka-3-6-6/doc/weka/core/pmml/MiningSchema.html]

MiningSchema


---


|  |  |  |
| --- | --- | --- |
| **Overview** \| **Package** \| **Class** \| **Tree** \| **Deprecated** \| **Index** \| **Help** \| **Weka's home** | |  |
| **PREV CLASS**   **NEXT CLASS** | **FRAMES**    **NO FRAMES**     **All Classes** |  |
| SUMMARY: NESTED \| FIELD \| CONSTR \| METHOD | DETAIL: FIELD \| CONSTR \| METHOD |  |


---


## weka.core.pmml Class MiningSchema

```
java.lang.Object
  weka.core.pmml.MiningSchema
```

**All Implemented Interfaces:**: java.io.Serializable

---

``` public class MiningSchema extends java.lang.Object implements java.io.Serializable ```

This class encapsulates the mining schema from
a PMML xml file. Specifically, it contains the
fields used in the PMML model as an Instances
object (just the header). It also contains meta
information such as value ranges and how to handle
missing values, outliers etc.
We also store various other PMML elements here, such as
the TransformationDictionary, DerivedFields and Targets
(if defined). They are not part of the mining schema per se, but
relate to inputs used by the model and it is convenient to
store them here.

**Version:**
:   $Revision: 5562 $

**Author:**
:   Mark Hall (mhall{[at]}pentaho{[dot]}com)

**See Also:**: Serialized Form

---

**Constructor Summary**

`MiningSchema(org.w3c.dom.Element model,
Instances dataDictionary,
weka.core.pmml.TransformationDictionary transDict)`
  
          Constructor for MiningSchema.


| **Method Summary** | |
| --- | --- |
| `void` | `applyMissingAndOutlierTreatments(double[] values)`             Apply both missing and outlier treatments to an incoming instance. |
| `void` | `applyMissingValuesTreatment(double[] values)`             Apply the missing value treatments (if any) to an incoming instance. |
| `void` | `applyOutlierTreatment(double[] values)`             Apply the outlier treatment methods (if any) to an incoming instance. |
| `void` | `convertNumericAttToNominal(int index, java.util.ArrayList<java.lang.String> newVals)`             Convert a numeric attribute in the mining schema to nominal. |
| `void` | `convertStringAttsToNominal()`             Method to convert any string attributes in the mining schema Instances to nominal attributes. |
| `java.util.ArrayList<DerivedFieldMetaInfo>` | `getDerivedFields()` |
| `Instances` | `getFieldsAsInstances()`             Get the all the fields (both mining schema and derived) as Instances. |
| `java.util.ArrayList<MiningFieldMetaInfo>` | `getMiningFields()` |
| `Instances` | `getMiningSchemaAsInstances()`             Get the mining schema fields as an Instances object. |
| `TargetMetaInfo` | `getTargetMetaData()`             Get the Target meta data. |
| `weka.core.pmml.TransformationDictionary` | `getTransformationDictionary()`             Get the transformation dictionary . |
| `boolean` | `hasTargetMetaData()`             Returns true if there is Target meta data. |
| `java.lang.String` | `toString()`             Get a textual description of the mining schema. |

**Methods inherited from class java.lang.Object**

`equals, getClass, hashCode, notify, notifyAll, wait, wait, wait`

**Constructor Detail**

### MiningSchema

```
public MiningSchema(org.w3c.dom.Element model,
                    Instances dataDictionary,
                    weka.core.pmml.TransformationDictionary transDict)
             throws java.lang.Exception
```

:   Constructor for MiningSchema.

    **Parameters:**: `model` - the `Element` encapsulating the pmml model: `dataDictionary` - the data dictionary as an Instances object **Throws:**: `java.lang.Exception` - if something goes wrong during construction of the mining schema


**Method Detail**

### applyMissingValuesTreatment

```
public void applyMissingValuesTreatment(double[] values)
                                 throws java.lang.Exception
```

:   Apply the missing value treatments (if any) to an incoming instance.

    :   **Parameters:**: `values` - an array of doubles in order of the fields in the mining schema that represents the incoming instance (note: use PMMLUtils.instanceToSchema() to generate this). **Throws:**: `java.lang.Exception` - if something goes wrong during missing value handling

---


### applyOutlierTreatment

```
public void applyOutlierTreatment(double[] values)
                           throws java.lang.Exception
```

:   Apply the outlier treatment methods (if any) to an incoming instance.

    :   **Parameters:**: `values` - an array of doubles in order of the fields in the mining schema that represents the incoming instance (note: use PMMLUtils.instanceToSchema() to generate this). **Throws:**: `java.lang.Exception` - if something goes wrong during outlier treatment handling

---


### applyMissingAndOutlierTreatments

```
public void applyMissingAndOutlierTreatments(double[] values)
                                      throws java.lang.Exception
```

:   Apply both missing and outlier treatments to an incoming instance.

    :   **Parameters:**: `values` - an array of doubles in order of the fields in the mining schema that represents the incoming instance (note: use MappingInfo.instanceToSchema() to generate this). **Throws:**: `java.lang.Exception` - if something goes wrong during this process

---


### getFieldsAsInstances

```
public Instances getFieldsAsInstances()
```

:   Get the all the fields (both mining schema and derived) as Instances.
    Attributes are in order of those in the mining schema, followed by
    derived attributes from the TransformationDictionary followed by
    derived attributes from LocalTransformations.

    :   **Returns:**: all the fields as an Instances object

---


### getMiningSchemaAsInstances

```
public Instances getMiningSchemaAsInstances()
```

:   Get the mining schema fields as an Instances object.

    :   **Returns:**: the mining schema fields as an Instances object.

---


### getTransformationDictionary

```
public weka.core.pmml.TransformationDictionary getTransformationDictionary()
```

:   Get the transformation dictionary .

    :   **Returns:**: the transformation dictionary or null if none is defined.

---


### hasTargetMetaData

```
public boolean hasTargetMetaData()
```

:   Returns true if there is Target meta data.

    :   **Returns:**: true if there is Target meta data

---


### getTargetMetaData

```
public TargetMetaInfo getTargetMetaData()
```

:   Get the Target meta data.

    :   **Returns:**: the Target meta data

---


### convertStringAttsToNominal

```
public void convertStringAttsToNominal()
```

:   Method to convert any string attributes in the mining schema
    Instances to nominal attributes. This may be necessary if there are
    no Value elements defined for categorical fields in the data dictionary.
    In this case, elements in the actual model definition will probably reveal
    the valid values for categorical fields.

---


### convertNumericAttToNominal

```
public void convertNumericAttToNominal(int index,
                                       java.util.ArrayList<java.lang.String> newVals)
```

:   Convert a numeric attribute in the mining schema to nominal.

    :   **Parameters:**: `index` - the index of the attribute to convert: `newVals` - an ArrayList of the values of the nominal attribute

---


### getDerivedFields

```
public java.util.ArrayList<DerivedFieldMetaInfo> getDerivedFields()
```

---


### getMiningFields

```
public java.util.ArrayList<MiningFieldMetaInfo> getMiningFields()
```

---


### toString

```
public java.lang.String toString()
```

:   Get a textual description of the mining schema.

    :   **Overrides:**: `toString` in class `java.lang.Object`
    :   **Returns:**: a textual description of the mining schema


---


|  |  |  |
| --- | --- | --- |
| **Overview** \| **Package** \| **Class** \| **Tree** \| **Deprecated** \| **Index** \| **Help** \| **Weka's home** | |  |
| **PREV CLASS**   **NEXT CLASS** | **FRAMES**    **NO FRAMES**     **All Classes** |  |
| SUMMARY: NESTED \| FIELD \| CONSTR \| METHOD | DETAIL: FIELD \| CONSTR \| METHOD |  |


---
